# Supplementary material for: Drug-target Mendelian randomization analysis supports lowering plasma ANGPTL3, ANGPTL4, and APOC3 levels as strategies for reducing cardiovascular disease risk
Source: Eur Heart J Open. 2024 Apr 30;4(3):oeae035. doi: 10.1093/ehjopen/oeae035 (PMC11182694; doi:10.1093/ehjopen/oeae035)
Supplement: oeae035_Supplementary_Data [file oeae035_supplementary_data.zip › online_supplemental_tables_1_10.docx]

**Supplemental Tables**

*The Supplemental Tables 1-10 are located within the supplemental_tables_1_10.xlsx file. The .xlsx file is available via a public repository and can be accessed via the following link:* <https://doi.org/10.5281/zenodo.11192765>
